# Low vision status and declining vision decrease Health-Related Quality of Life: Results from a nationwide 11-year follow-up study

**DOI:** 10.1007/s11136-019-02260-3

**Published:** 2019-08-10

**Authors:** Joonas Taipale, Alexandra Mikhailova, Matti Ojamo, Janika Nättinen, Saku Väätäinen, Mika Gissler, Seppo Koskinen, Harri Rissanen, Päivi Sainio, Hannu Uusitalo

**Affiliations:** 1 Faculty of Medicine and Health Technology, Department of Ophthalmology, University of Tampere, Tampere, Finland; 2 Finnish Register of Visual Impairment, Finnish Federation of the Visually Impaired, Helsinki, Finland; 3 National Institute for Health and Welfare, Helsinki, Finland; 4 ESiOR Ltd, Kuopio, Finland; 5 School of Pharmacy, University of Eastern Finland, Kuopio, Finland; 6 Department of Neurobiology, Care Sciences and Society, Karolinska Institute, Stockholm, Sweden; 7 Tays Eye Center, Tampere University Hospital, Tampere, Finland

**Keywords:** Quality of life, Visual acuity, Follow-up study, Population-based study, Epidemiology

## Abstract

**Purpose:**

The impact of visual acuity (VA) on Health-Related Quality of Life (HRQoL) and the cross-sectional and longitudinal differences in HRQoL during the 11-year follow-up were investigated. The aim was to examine the impact declining vision has on HRQoL and to provide comparable data to facilitate the allocation of health-care resources.

**Methods:**

We utilized nationwide health examination surveys carried out by the National Institute for Health and Welfare in 2000 and 2011, providing a representative sampling of the Finnish adult population aged 30 and older. VA was assessed through Snellen E test, and HRQoL scores were evaluated using EQ-5D and 15D questionnaires. Multiple imputations with Markov chain Monte Carlo method was used to utilize the data more effectively. Regression analyses were conducted to assess the impact of declining VA on HRQoL, adjusted for incident comorbidities.

**Results:**

Lower VA status was associated with significantly lower HRQoL at both time points, most clearly observable below the VA level of 0.5. Declining VA resulted in statistically significant decline in HRQoL during the follow-up, greater with distance than near VA. 15D impairment associated with decline in the distance VA was also clinically meaningful and greater than that associated with any of the examined comorbidities.

**Conclusions:**

HRQoL was significantly and meaningfully impaired even before the threshold of severe vision loss or blindness was reached. The results encourage the improvement of available treatment options aiming to postpone the onset of visual impairment or declining VA, to maintain better quality of life among the population.

**Electronic supplementary material:**

The online version of this article (10.1007/s11136-019-02260-3) contains supplementary material, which is available to authorized users.

## Introduction

Vision plays a major role in maintaining the abilities needed in everyday life, such as learning, working capacity, self-care, and mobility [1–3]. For instance, Gompel et al. report, that children with low visual acuity (VA) need more time for reading and comprehending text, while the comprehension skill itself does not differ from those with normal VA. [4] Impaired VA is associated with increased risk of accidents, particularly falling [5]. Along with maintaining the functional ability, there is evidence suggesting poor vision increases the risk of institutionalization [6]. Declining vision also causes problems in different aspects of daily life, for instance, in social interactions and daily routines [7–9]. In Finland, visually impaired people have a lower level of education and employment on average, compared to the overall population [10].

Some of the other population-based studies previously conducted focusing on vision and declining VA are The Rotterdam Study [11], The Blue Mountain Eye Study [12], Korea National Health and Nutrition Examination Survey [13], Melbourne Visual Impairment Project [14], and The Beaver Dam Eye Study [15]. Being researched widely around the globe, none of these, however, include Health-Related Quality of Life (HRQoL) and analysis of its connection to declining VA.

Most of the previously conducted research on Quality of Life (QoL) and vision is highly focused only on the visually impaired or on specific eye diseases and their impact on QoL [16–18]. Therefore, they tend to be based on relatively small study populations that are not representative on a larger scale. Furthermore, eye diseases may also have an impact on QoL through other factors besides declining VA, such as potential adverse effects of medication, anxiety about the future, lost time and money spent on the diagnostics, therapeutic interventions, and follow-up, which may cause bias [19, 20]. To our knowledge, there is only one study investigating the impact of declining visual function on HRQoL in general population—a longitudinal population-based study among Latino people [21], but the generalizability of these results into other ethnicities is currently unknown.

Correlation of declining VA and QoL has been studied to some extent using vision-specific QoL assessments, such as the Vision-Related Quality of Life (VRQoL) questionnaire [21, 22]. These instruments are considered highly condition specific [23], not allowing comparison across diseases and/or treatments. For generalizability and comparability, generic preference-based questionnaires are required. Quality of life measured through VRQoL—questionnaire might also be influenced by non-visual factors [24]. Hence, it is important to contribute information about declining VA regardless of eye diseases.

In this study, we examined the profiles and the correlation of VA and self-reported HRQoL in Finnish adults in two time points in 2000 and 2011 using nationwide population-based health surveys and generic preference-based HRQoL tools. This comprehensive approach allows us to get an overall perspective on QoL and how it is affected by vision loss. Using generic tools instead of vision-specific measures reduces the potential reporting bias associated with overemphasis of visual factors on the responders’ QoL.

## Methods

### Study population and survey design

We utilized data from two nationwide surveys of health and well-being carried out by the National Institute for Health and Welfare in 2000 and 2011. These Health 2000 and 2011 studies provided a probability-clustered sampling and weighting scheme that estimates health statistics that are representative of Finnish adult population aged 30 and older at the time of sampling. The sampling scheme also accounts for designed oversampling among the elderly people in the 2000 baseline. Our sample inclusively represents the Finnish adult population with respect to main demographics of Finland. The general research methods have previously been described elsewhere in more detail [25, 26]. Briefly, the study participants were invited to participate in an interview and health examination in 2000, and in a follow-up survey in 2011. The demographics of study participants are summarized in Table 1.

**Table 1:** The demographics of study participants aged 30 years or older

	2000	2011	Both time points
Sample size (% women)	8028 (54.7%)	8006 (53.0%)	4703 (55.5%)
Mean age (SD)	54.71 (16.2)	55.34 (15.6)	49.6 (12.1)^a^ 60.0 (12.1)^b^
EQ-5D Index Score available	6148	4084	3131
15D index score available	6166	4266	3510
Distance VA measured	6674	4619	3867
Near VA measured	6646	4618	3860

^a^In baseline

^b^In follow-up

The mean age for those with complete data on VA was 49.6 years in 2000 and 60.1 years in 2011 and the proportion of women was 55.3% (not shown in the table) in both time points, following quite well the general study population distributions

### Visual acuity tests

Habitual distance VA was measured binocularly at 4 m, with current visual correction, using the Snellen eye chart. Habitual near VA was measured at the participant’s preferred reading distance, using the near vision chart. Illumination was set to ≥ 350 lx on the vision charts. [25, 26] All VA values are presented as decimal equivalents. For comparisons, the VA values were further classified based on forthcoming ICD-11 classification [27] and a priori judgement based on the clinical relevance of VA. VA ≥ 1.0 was classified as good vision, VA of 0.63–0.8 as adequate vision, VA ≤ 0.5 as weak vision, VA ≤ 0.25 as impaired vision, and VA < 0.1 as severe vision loss or blindness.

Habitual distance and near VA were measured at both time points (2000 and 2011). A change of at least two lines on the Snellen eye chart was considered clinically significant improvement or decline, as smaller changes can be caused by numerous other factors, such as state of the tear film on ocular surfaces or preceding fatigue causing accommodative tiredness during the measurement [28–30].

### Health-Related Quality of Life assessment

HRQoL was assessed using two internationally established, generic and standardized preference-based questionnaires—EQ-5D and 15D [31, 32]. Both methods yield a single index score, as well as a multidimensional profile. The EQ-5D has an answer scale ranging from 1 (no difficulties) to 3 (extreme difficulty) and consists of five dimensions: mobility, self-care, usual activities, pain/discomfort, and anxiety/depression. The 15D has an answer scale ranging from 1 (no difficulties) to 5 (extreme difficulty) and comprises 15 dimensions: mobility, vision, hearing, breathing, sleeping, eating, speech, excretion, usual activities, mental function, sexual activity, discomfort and symptoms, depression, distress, and vitality. For both methods, weighting the separate dimensions with population-based preference weights yields index scores ranging from 0 to 1 for 15D and from − 0.59 to 1 for EQ-5D, with 1 representing the best possible HRQoL. In the present study, 15D was weighted using Finnish preference weights, whereas EQ-5D was weighted using UK time-trade-off weights in order to achieve the widest possible comparability [33]. Clinically meaningful difference can be defined as the least change health-care professionals or the study participants themselves may observe. In the present study, we used previously given thresholds of ≥ 0.07 for EQ-5D and ≥ 0.015 for 15D as clinically meaningful differences [34, 35].

For the examination of separate dimensions of EQ-5D, dimension level 1 was considered as “no difficulties” while reported level 2–3 resulted “difficulties” in such dimension. For 15D, the individual dimensions were converted to 0–1 scale by applying the established Finnish multi-attribute utility weights. Validity, feasibility, and reliability of this method are further discussed in previous publication. [36] For brevity, only ‘Vision’ dimension of 15 total individual dimensions included in 15D was reported separately here in addition to 15D index scores.

HRQoL was evaluated in both time points (2000 and 2011) to assess the changes during the follow-up period. Eye examination alongside the HRQoL assessment made it possible to evaluate the effect of declining vision on the quality of life.

### Data analysis

The overall prevalence for distance and near VA grouped according to age was estimated by using population data at StatFin database (Statistics Finland). The data (collected and reported annually) were applied for both samples according to the investigation year.

Odds ratios for individual EQ-5D dimensions and correlations between measured distance or near VA and HRQoL index scores were conducted implementing the Complex Samples module in IBM SPSS Statistics for Windows, version 24 (IBM Corp., Armonk, N.Y., USA), to account for the complex sampling design. The analyses were adjusted for age, sex, and the most common comorbidities, specified below. P-values were adjusted with Bonferroni correction when making multiple comparisons. Changes in VA during the follow-up period and its impact on HRQoL changes were estimated through linear regression with adjustments for new incident diagnoses of the comorbidities, as well as baseline HRQoL. Variance inflation factors (VIFs) were used to measure multicollinearity in regression analyses.

To utilize the data more effectively, multiple imputations method was used to handle missing comorbidities in the regression analyses concerning the longitudinal changes [37]. Missing data were predicted using respondent’s non-missing data in five imputations applying iterative Markov chain Monte Carlo method [38]. After conducting the five imputations, the estimates of the variables with previously missing values were pooled to give single estimates to be utilized in the final analysis. Missing VA changes were not imputed.

When comparing the data between the time points, the weighting scheme calculated by National Institute for Health and Welfare was applied to account for the intentional oversampling in 2000 time point as well as the loss to follow-up. The sampling scheme is based on IPW-method (reverse probability), further discussed in the previous publications. [39, 40] Subgroups with minimum size of three participants were included in population analysis. Age distributions for both time points, taken from population statistics in the StatFin (Statistics Finland) database, were applied to better represent the impact of declining VA population-wise. Kendall’s tau-B test and/or regression models were used when estimating the associations between continuous and ordinal variables.

### Comorbidities

For most of the analyses, common diseases were considered to account for their potential impact on the HRQoL. The diseases were self-reported both in 2000 and 2011 and were classified to major comorbidity groups for robustness. Myocardial infarction, Angina Pectoris, heart failure, rhythm disorders, and “other heart disorder” were considered as “Heart diseases.” Asthma, chronic obstructive pulmonary disease (COPD), chronic bronchitis, and “other pulmonary disease” were categorized as “Pulmonary diseases.” “Vascular diseases” included stroke and varicose veins in lower limbs. “Musculoskeletal conditions” included self-reported rheumatoid arthritis, arthrosis, fractures, and osteoporosis. “Psychiatric diseases” consists of psychotic disorders, depression, anxiety, psychoactive substance abuse, or “other psychiatric disease.” In addition, hypertension, diabetes, Parkinson’s disease, and cancer (unspecified) were included in our model.

Study participant was considered to have comorbidity, if participant reported having any of the conditions included in the comorbidity group. When examining new incident diagnoses during the follow-up period, each condition was scrutinized in 2000 baseline and in 2011 follow-up. If study participant reported one or more new condition included in the given comorbidity group during 2011 follow-up, participant was classified as having incident comorbidity, regardless of the presence of other conditions included in that specific comorbidity group in baseline.

## Results

### Visual acuity in the study population

The proportion of participants with good VA decreased by age at both time points (Table 2). Average distance and near VA improved between the time points, and the age-, and sex-adjusted prevalence of good distance and near VA increased. The cross-sectional correlation between the VA and HRQoL index values was strongest for VA below 0.5, as shown in our Supplementary Figs. S1 and S2.

**Table 2:** Distance and near VA in different age groups in Health 2000 and 2011 studies

	2000						2011					
	30–44	45–54	55–64	65–74	75+	All	30–44	45–54	55–64	65–74	75+	All
*Distance vision, n*	2210	1666	1124	842	832	**6674**	1039	1057	1160	850	513	**4619**
*Women* *%*	52.9	51.3	53.5	56.8	70.6	**55.2**	57.4	55.0	52.8	55.5	59.3	**55.5**
Good % (VA ≥ 1)	94.0	85.0	78.7	53.7	17.9	**76.7**	95.6	91.0	83.8	70.5	31.2	**80.0**
Adequate % (VA 0.63**–**0.8)	4.4	12.0	16.5	34.0	39.9	**15.7**	3.5	7.3	12.4	23.3	43.2	**14.4**
Weak % (VA 0.32–0.5)	1.5	2.8	4.3	9.9	29.4	**6.0**	0.8	1.4	2.8	4.6	19.7	**4.3**
Impaired % (0.1–0.25)	0.1	0.1	0.4	1.8	8.3	**1.1**	0.2	0.2	0.8	1.1	4.3	**1.0**
Severe loss % (VA < 0.1)	0.0	0.2	0.2	0.6	4.5	**0.6**	0.0	0.0	0.2	0.5	1.6	**0.3**
*Average distance VA*	1.16	1.09	1.03	0.88	0.58	**1.01**	1.19	1.14	1.08	0.99	0.74	**1.07**
*Near vision (n)*	2 210	1 661	1 118	838	819	**6 646**	1 039	1 055	1 160	849	515	**4 618**
*Women* *%*	52.9	51.4	53.5	56.7	70.8	**55.3**	57.4	54.9	52.8	55.5	59.4	**55.5**
Good % (VA ≥ 1)	86.4	54.5	56.3	42.7	18.8	**60.8**	90.0	63.7	61.9	53.3	28.7	**64.3**
Adequate % (VA 0.63–0.8)	12.4	37.7	37.3	45.8	47.5	**31.0**	8.9	31.8	31.6	38.3	47.2	**28.7**
Weak % (VA 0.32–0.5)	1.1	6.8	5.7	9.4	22.0	**6.4**	1.1	4.1	4.4	7.1	18.8	**5.6**
Impaired % (0.1–0.25)	0.2	0.9	0.5	2.0	10.1	**1.6**	0.0	0.3	1.9	0.9	4.5	**1.2**
Severe loss % (VA < 0.1)	0.0	0.1	0.2	0.0	1.5	**0.2**	0.0	0.0	0.2	0.5	0.8	**0.2**
*Average near VA*	1.14	0.92	0.92	0.84	0.63	**0.95**	1.16	0.99	0.96	0.91	0.75	**0.99**

The prevalence is adjusted based on the weighting scheme of the National Institute for Health and Welfare. Different weights have been applied for 2000 and 2011 data to represent the Finnish population in each of these time points. Weighting is adjusted for age and sex, and also accounts for the loss between the time points

### Cross-sectional association between vision and health-related quality of life

**Fig. 1:**
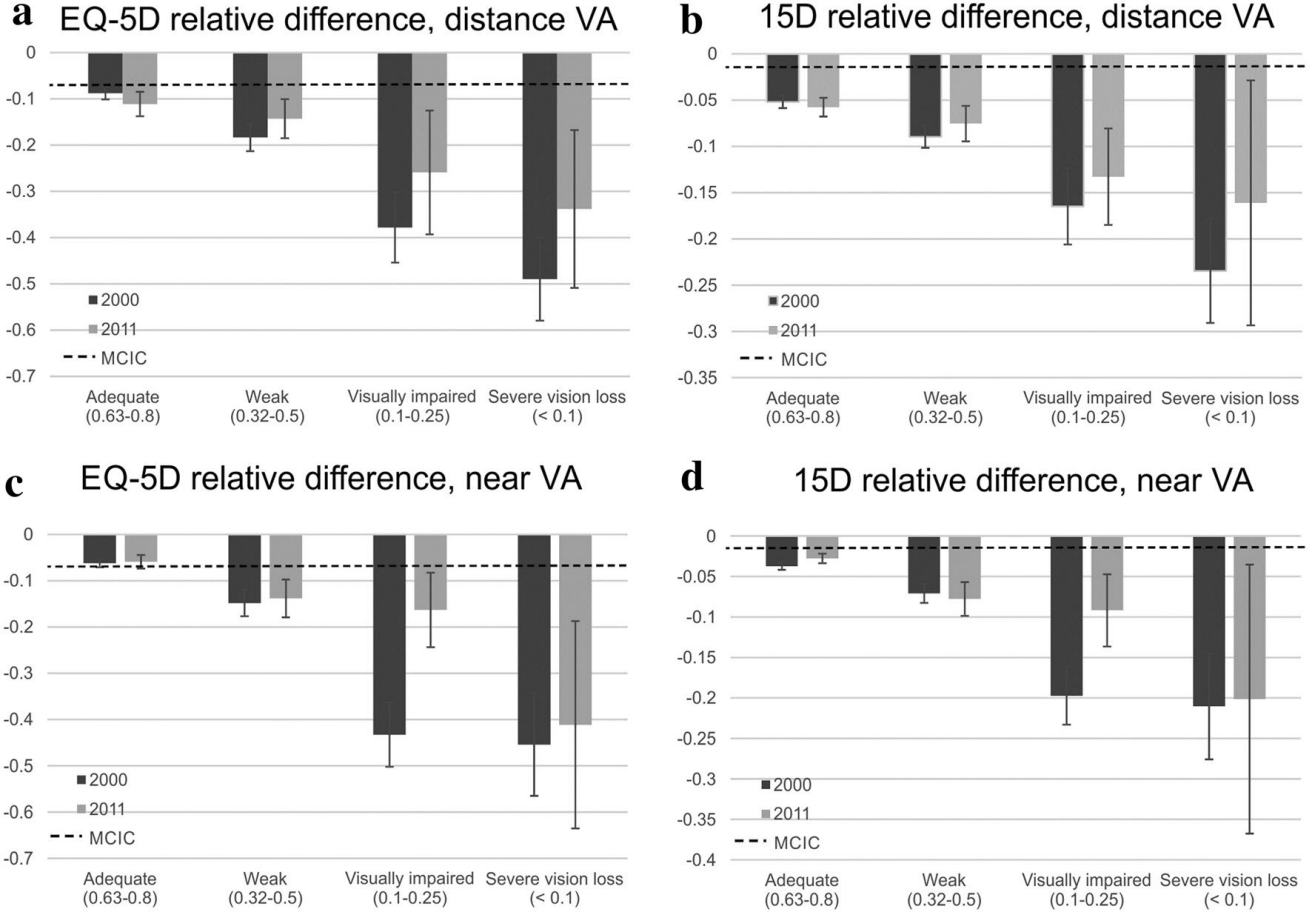
Differences in Health-Related Quality of Life (HRQoL) index scores in relation to those with good habitual distance (**a**, **b**) and near (**c**, **d**) visual acuity (VA) in both time points. The *y*-axis represents the mean index value difference in relation to good VA (VA ≥ 1.0). The *x*-axis represents VA groups. Dashed line represents the minimal clinically important change (MCIC), 0.07 for EQ-5D and 0.015 for 15D [34, 35]. The weighting scheme is applied to address the differences in study populations’ age and sex distributions and to allow the comparison between the time points. The mean HRQoL index values for good VA group were 0.87 for **a**, 0.93 for **b**, 0.88 in 2000, and 0.87 in 2011 for **c** and 0.93 for **d**

The differences between study participants with good (VA ≥ 1.0) and weak distance vision (threshold of VA ≤ 0.5, LogMAR 0.3) were statistically significant (*p* < 0.001) with both EQ-5D (Fig. 1a) and 15D (Fig. 1b), suggesting that lower vision status associates with declining quality of life even before it reaches the threshold of visual impairment or blindness. The trends were similar for near vision, with statistically significant differences observed (*p* < 0.001) with both HRQoL instruments (Fig. 1c, d) and clinically meaningful for 15D assessment results even in adequate VA group (Fig. 1b, d).

**Fig. 2:**
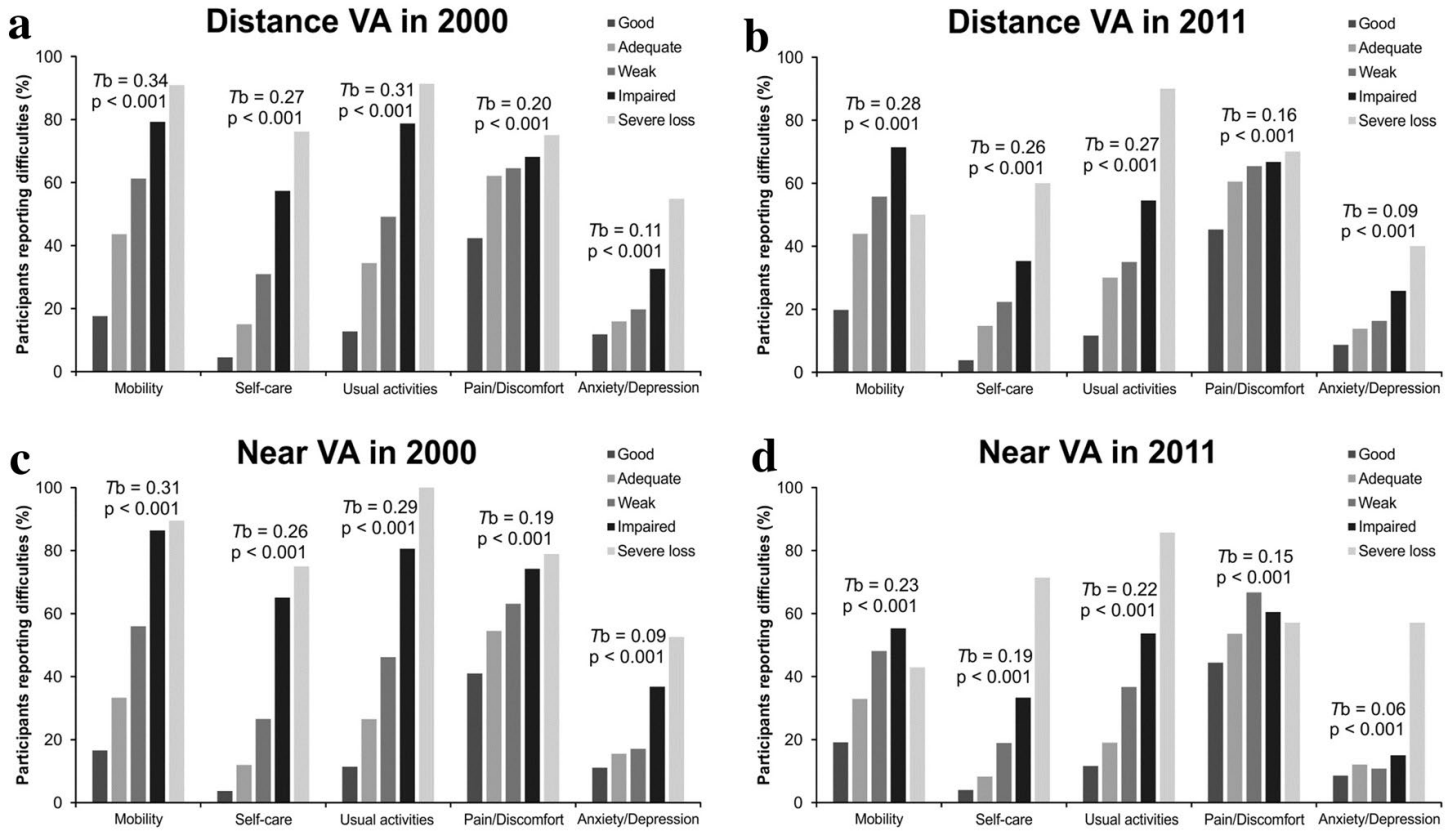
The proportion of participants reporting difficulties in individual EQ-5D dimensions, grouped according to visual acuity (VA). The *x*-axis represents EQ-5D dimensions for each VA group and the *y*-axis the proportion of participants reporting difficulties (answering 2 or 3 to a dimension). Correlations according to Kendall’s tau-B with their *p* values are shown for each dimension. All values presented are adjusted for sex and age

When considering the individual HRQoL dimensions, the most notable correlation with lower distance or near VA statuses was observed for mobility, self-care, and usual activities (Fig. 2). Interestingly, anxiety/depression EQ-5D dimension, while statistically significant, appeared to have only relatively weak association with lower VA groups.

After adjusting for sex, age, and multiple comorbidities, the increasing proportion of respondents reported difficulties in every EQ-5D dimension except for pain/discomfort dimension with declining distance VA in 2000 (Table 3). Most of the Odds Ratios, compared to those with good VA, were also statistically significant. At the follow-up assessment, the increasing odds were statistically significant only for usual activities dimension. The odds of having difficulties especially in the EQ-5D dimensions of usual activities and self-care clearly increased compared to those with good VA, particularly when examining those with lower near VA (Table 4). The differences in associations between the time points were clearly visible in the dimensions of mobility and self-care, especially when examining correlation between near VA and these EQ-5D dimensions. In the study population, having difficulties concerning anxiety/depression seemed to become prominent only when impairment or severe vision loss was observed. This was the case especially in 2000, when the odds for experiencing problems were over threefold. In 2011, the impact of declined VA on anxiety/depression was statistically insignificant. In baseline, declined VA seemed to have a broader impact on the EQ-5D dimensions, having significant effect on four dimensions (all except pain/discomfort), while in 2011 only one dimension for distance VA (usual activities) and two dimensions for near VA (usual activities and self-care) were statistically significantly affected. Usual activities were the EQ-5D dimension most strongly associated with declined VA. Being statistically significant in both time points for near and distance VA, it increases the OR to as high as tenfold comparing to those with good VA.

**Table 3:** The odds of having difficulties in EQ-5D dimensions compared to those in good distance visual acuity (VA ≥ 1.0) group

2000 Distance VA	Mobility (*p* = 0.001)	Self-care (*p* < 0.001)	Usual activities (*p* < 0.001)	Pain/discomfort (*p* = 1.000)	Anxiety/depression (*p* < 0.001)
Adequate 0.63–0.8	1.173 (0.991–1.389)	1.217 (0.951–1.557)	**1.474 (1.242–1.748)**	1.141 (0.959–1.359)	1.051 (0.847–1.304)
Weak 0.32–0.5	**1.670 (1.256–2.221)**	**2.240 (1.617–3.103)**	**2.044 (1.543–2.707)**	0.910 (0.712–1.163)	1.287 (0.916–1.808)
Impaired or severe loss ≤ 0.25	**2.805 (1.601–4.915)**	**6.612 (4.137–10.568)**	**9.149 (5.172–16.183)**	0.967 (0.555–1.683)	**3.625 (2.235–5.881)**

2011 Distance VA	Mobility (*p* = 0.547)	Self-care (*p* = 0.143)	Usual activities (*p* < 0.001)	Pain/discomfort (*p* = 1.000)	Anxiety/depression (*p* = 0.077)
Adequate 0.63–0.8	**1.436 (1.124–1.833)**	**1.712 (1.131–2.589)**	**1.757 (1.340–2.304)**	1.237 (0.978–1.566)	**1.539 (1.062–2.230)**
Weak 0.32–0.5	**1.604 (1.036–2.484)**	**2.280 (1.282–4.055)**	1.297 (0.785–2.142)	1.192 (0.758–1.873)	**1.937 (1.077–3.483)**
Impaired or severe loss ≤ 0.25	2.100 (0.705–6.249)	**3.223 (1.013–10.254)**	**8.702 (3.465–21.851)**	1.109 (0.437–2.810)	**2.525 (1.118–5.700)**

The Odds Ratios (with 95% CI) are estimated through SPSS complex samples logistic regression analysis adjusted with sex, age, and the following comorbidities: heart diseases, pulmonary diseases, vascular diseases, musculoskeletal conditions, psychiatric diseases, hypertension, diabetes, Parkinson’s disease, and cancer. *p* values, showing the trends, are adjusted with Bonferroni correction for multiple comparisons. Bolded values denote statistically significant (*p* < 0.05) odds ratios compared to good vision (VA ≥ 1.0)

**Table 4:** The odds of having difficulties in EQ-5D dimensions compared to those in good near visual acuity (VA ≥ 1.0) group

2000 near VA	Mobility (*p* < 0.001)	Self-care (*p* < 0.001)	Usual activities (*p* < 0.001)	Pain/discomfort (*p* = 0.458)	Anxiety/depression (*p* < 0.001)
Adequate 0.63–0.8	1.158 (0.991–1.354)	**1.695 (1.325**–**2.167)**	**1.475 (1.255**–**1.735)**	1.076 (0.956–1.210)	**1.242 (1.021**–**1.510)**
Weak 0.32–0.5	**1.989 (1.491**–**2.652)**	**2.736 (1.922**–**3.894)**	**2.481 (1.838**–**3.348)**	1.126 (0.869–1.460)	1.045 (0.696–1.570)
Impaired or severe loss ≤ 0.25	**6.803 (3.863**–**11.980)**	**9.811 (6.161**–**15.622)**	**10.279 (5.923**–**17.839)**	1.499 (0.861–2.612)	**3.471 (2.014**–**5.980)**

2011 near VA	Mobility (p = 0.646)	Self-care (p = 0.023)	Usual activities (p < 0.001)	Pain/discomfort (p = 1.000)	Anxiety/depression (p = 0.509)
Adequate 0.63–0.8	1.207 (0.991–1.470)	1.019 (0.712–1.459)	1.088 (0.840–1.408)	1.035 (0.885–1.210)	1.295 (0.940–1.785)
Weak 0.32–0.5	**1.759 (1.185**–**2.611)**	**2.039 (1.078**–**3.857)**	**2.449 (1.589**–**3.775)**	**1.771 (1.179**–**2.660)**	1.095 (0.621–1.932)
Impaired or severe loss ≤ 0.25	1.620 (0.755–3.477)	**3.650 (1.410**–**9.453)**	**4.652 (2.249**–**9.622)**	1.154 (0.530–2.515)	2.023 (0.740–5.533)

The Odds Ratios (with 95% CI) are estimated through SPSS complex samples logistic regression analysis adjusted with sex, age, and the following comorbidities: heart diseases, pulmonary diseases, vascular diseases, musculoskeletal conditions, psychiatric diseases, hypertension, diabetes, Parkinson’s disease, and cancer. *p* values, showing the trends, are adjusted with Bonferroni correction for multiple comparisons. Bolded values denote statistically significant (*p* < 0.05) odds ratios compared to good vision (VA ≥ 1.0)

### Changes in visual acuity and Health-Related Quality of Life during 11 years

The results of the multivariable regression analysis examining the associations between changes in HRQoL index values and changes in VA are presented in Table 5. When adjusted for the incidence of common comorbidities, association between decline in both distance VA and near VA was statistically significantly associated with declines in both EQ-5D and 15D over the 11-year study period, although the association with declining distance VA was greater than with declining near VA. Decline in distance VA was associated also with clinically meaningful decline in 15D. Findings did not change substantially, when only statistically significant (*p* < 0.05) factors were included as explanatory variables regression model in stepwise-insertion analysis (see Supplementary Table S1). Multicollinearity was tested through VIFs (Variance inflation factors), which ranged from 1.007 to 1.147 for the variables included in the models, denoting no or very little multicollinearity.

**Table 5:** Multivariable regression analysis examining the changes in EQ-5D and 15D index values between 2000 and 2011

	Change in EQ-5D (*n* = 3068)		Change in 15D (*n* = 3454)	
	B coefficients	Beta coefficients	B coefficients	Beta coefficients
Constant	0.409**		0.272**	
Male sex	+ 0.010	+ 0.029	− 0.002	− 0.013
Incident heart disease	− 0.032*	− 0.054*	− 0.015**	− 0.065**
Incident pulmonary disease	− 0.024*	− 0.034*	− 0.024**	− 0.086**
Incident vascular disease	− 0.013	− 0.017	− 0.013*	− 0.043*
Incident musculoskeletal condition	− 0.038**	− 0.103**	− 0.007*	− 0.047*
Incident hypertension	− 0.029**	− 0.064**	− 0.005	− 0.027
Incident diabetes	− 0.014	− 0.020	− 0.017**	− 0.061**
Incident psychiatric disorder	− 0.060**	− 0.069**	− 0.024**	− 0.070**
Incident Parkinson disease	− **0.071**	− 0.026	− 0.077**	− 0.066**
Incident cancer	− 0.021	− 0.028	− 0.012*	− 0.041*
Change in Visual acuity (VA), compared to stable VA				
Distance VA declined	− 0.062**	− 0.090**	− 0.033**	−0.117**
Distance VA improved	− 0.028	− 0.027	+ 0.000	+ 0.000
Near VA declined	− 0.028*	− 0.049*	− 0.012*	− 0.052*
Near VA improved	− 0.010	− 0.012	+ 0.005	+ 0.014
QoL index value in baseline	− 0.473**	− 0.427**	− 0.293**	− 0.312**
*R*^2^	0.197**	0.193**	0.132**	0.128**

VA was considered improved or declined if difference of at least 2 lines in the Snellen eye chart was observed between the time points. The unstandardized B coefficients show the magnitude of the impact on HRQoL, while the standardized Beta coefficients allow the comparison of the explanatory variables with each other. Clinically meaningful B coefficients are bolded (≥ 0.07 for EQ-5D and ≥ 0.015 for 15D [34, 35]). It should be noted that B regression coefficients represented in the table are independent and additive, meaning that if an individual experience a, e.g., decline in both near and distance VA, the HRQoL impacts of both need to be considered (added together)

*Denotes statistical significance with *p* < 0.05

**Denotes statistical significance with *p* < 0.001

Newly diagnosed heart or pulmonary diseases were statistically significantly associated with the change in 15D vision dimension (Tables 6, S2). Naturally, declining near and distance VA also negatively affected the vision dimension value. The impact of declining distance VA seems to be somewhat greater than that of declining near VA. Interestingly, improved VA did not have a statistically significant association with the change in 15D vision dimension.

**Table 6:** Regression analysis examining the change in 15D vision dimension between 2000 and 2011

	Beta coefficients	Sig.
Constant	0.685	< 0.001
Male sex	− 0.014	0.344
Incident heart disease	− 0.010	0.484
Incident pulmonary disease	− 0.038	0.008
Incident vascular disease	+ 0.007	0.637
Incident musculoskeletal condition	+ 0.002	0.889
Incident hypertension	− 0.025	0.094
Incident diabetes	− 0.027	0.066
Incident psychiatric disorder	− 0.004	0.801
Incident Parkinson disease	− 0.016	0.248
Incident cancer	− 0.006	0.693
Change in visual acuity (VA), compared to stable VA		
Distance VA declined	− 0.190	< 0.001
Distance VA improved	− 0.008	0.553
Near VA declined	− 0.151	< 0.001
Near VA improved	− 0.004	0.788
15D Vision dimension value in baseline	− 0.493	< 0.001
Adjusted *R*^2^	0.301	< 0.001

VA was considered improved or declined if difference of at least 2 lines in the Snellen eye chart was observed between the time points. Only the standardized Beta coefficients reported to allow the comparison between the explanatory variables

## Discussion

Visual acuity plays a major role in self-reported HRQoL, the threshold of 0.5 being notable. Subjects with VA below this threshold have greater and progressive decreases in their HRQoL. Functionally, the threshold of 0.5 in distance VA is relevant; as with current visual correction, it serves as the requirement for standard driving license in many states in the United States of America [41] and the countries of the European Union (directive 2006/126/EC), thereby affecting individual’s activities of daily living. Moreover, previous publication reports differences in self-care when VA is near the threshold of 0.5 (from 0.4 to 0.63) [42]. Our results point out that low vision correlates with low HRQoL. The impact of having lower VA is also clinically meaningful and does not impact only single dimension of the assessments applied.

The odds of having difficulties in individual EQ-5D dimensions become greater as VA is lower, which is the most evident in the areas of self-care and usual activities. Those with weak VA are more than twice as likely to experience problems in those two dimensions, possibly increasing the need for daily assistance, compared to those with good VA. With VA being impaired or study participants blind, the odds progressively accumulate to over tenfold.

Visual acuity plays a major role in self-reported HRQoL, especially in the dimensions of usual activities and self-care. The magnitude of association between EQ-5D dimensions associated and the lower VA status decreased between the time points. Especially, for mobility, the impact of low VA is evident in baseline, while in 2011 the impact is no longer statistically significant. During this period, improved mobility aids, such as the Segway Personal Transporter, electric travel aids including GPS-locating, and tactile gloves have been implemented, which might have contributed for those with vision loss not experiencing difficulties with mobility, self-care, and usual activities [43–46]. Moreover, voice-controlled applications and devices have become routinely used. Environmental architecture and paying attention to accessibility may also contribute to the improvement, although there are still many aspects that complicate usual activities for those with visual disability [46]. Observed improvement in anxiety/depression dimension of EQ-5D between the time points may be partly due to improvements in availability and accessibility of social services between 2000 and 2011.

Low VA was associated with increased Anxiety/depression in the present study, and this has also been shown in previously conducted research [8, 47]. In our study, the impact of lower VA status in observed HRQoL became prominent only once vision was impaired or severely lost. While not in focus of the present study, in a supplemental analysis we examined the relation between anxiety/depression and measured VA, using Beck Depression Inventory (BDI), which purely concentrates on evaluating depression and mental well-being (see Supplementary Data). Unlike with anxiety/depression dimension of EQ-5D, where declining vision appears to have an impact only once vision is impaired or severely lost, BDI-scores and VA seem to have a linear connection. This promotes the endeavor to maintain visual function even before the onset of visual impairment. Due to the differences in questionnaires, EQ-5D is not as sensitive in identifying the early signs of anxiety/depression, applying also for 15D assessment. There may also be a psychological explanation for this, which is related to the question layout. In EQ-5D, the question about anxiety/depression is more straightforward with a limited answer scale, and study participants might not always identify depression and anxiety correctly. In BDI on the other hand, the subject is presented with a wide range of questions about the various, specific background factors contributing to depression, giving a more thorough analysis of the subject’s mental state.

The longitudinal findings from 15D assessment show similar changes in the index values. An important difference between 15D and EQ-5D assessments is that 15D includes a Vision dimension, which had a strong statistically significant association with declining VA in our analyses. Additionally, the decline is more strongly associated with a decline in distance VA than with a decline in near VA. Overall, our findings emphasize the importance of maintaining good VA to prevent the incremental loss of HRQoL.

Both our results and those of The Los Angeles Latino Eye Study (LALES) indicate that HRQoL, while evaluated with different assessments, shows significant correlation to declining vision, suggesting that aging alone does not account for the decline in HRQoL [21]. The LALES-study, while being a population-based study, unfortunately had a follow-up time of only 4 years. As a result, there were only 83 individuals whose VA declined in this time. These individuals appeared to have a slightly milder decrease (though statistically insignificant) in their general health (assessed via The National Eye Institute Visual Function Questionnaire (NEI VFQ-25)) when compared to those with stable VA. This is contrary to our findings, where declining VA caused decrease in HRQoL index values except for near VA applying EQ-5D assessment. For declining distance VA, HRQoL change assessed through 15D was also clinically meaningful. These findings may differ due to the differences in methods, follow-up time, study populations, or used HRQoL questionnaires.

The strengths of this study include a relatively long follow-up period of 11 years and a large study sample, which inclusively represents Finnish adult population aged 30 or older. Being widely collected and comprehensive, our study population and design reduced the impact of confounding factors. In comparison to samples collected from health-care units, our data do not consist of specific patient groups, which is a major strength.

Previously conducted research in German population-based sample confirms that VRQoL declines with age, assessed through NEI VFQ-25 questionnaire [22]. Their approach, however, lacks longitudinal perspective thereby making it difficult to exclude the impact of aging from other changes in society during the lifetime of different age groups. In our study, we took into account the effect of aging and common comorbidities, thus reducing their impact on the results.

As internationally established questionnaires were implemented in assessing the HRQoL, this study is comparable to previous and future research about conditions unrelated to vision. Questionnaire-based data collection represents the study participants’ perception of their everyday QoL. EQ-5D might not always be sensitive enough to show statistically significant changes as reported by Jones et al. [48]. However, when showing statistically significant differences, the specificity of this finding is evident in various health conditions [49–51].

The data collection was well-executed and a high proportion of the subjects in 2000 study participated also in the follow-up study in 2011. Although previously reported in other studies [52, 53], there were no significant differences in continuation in present study between males and females. Overall, the adherence to present study (58%) can be regarded good and in line with the previous studies, especially considering the trend of reduced participation in epidemiological studies during the past decades [54, 55]. Loss to follow-up was also compensated by applying calibrated weighting scheme and the differences in samples were adjusted by weighting the analyses with IPW-method. [39] Due to the study design, immigration to Finland after year 2000 has not been covered as the baseline sample defines characteristics for the weighting [26]. Study participants participating in the eye examination in 2000 were similar in their distribution when compared to those who had complete information from both time points.

When examining the association between longitudinal changes in HRQoL and VA, we accounted for incidence of common comorbidities. We found that 15D and EQ-5D were more strongly associated with the declining distance VA than with any of the examined incident comorbidities, except musculoskeletal conditions when examining EQ-5D. Association between HRQoL and declining near was not as large. The absolute impact of Parkinson’s disease on HRQoL was somewhat higher than impact of declining distance VA.

Saarni et al. have previously reported impact of various health conditions on HRQoL [56]. In their cross-sectional analysis, cataract and glaucoma have only marginal effect while the impact of macular degeneration is statistically significant and 15D-wise clinically important as well. However, in their analysis, health conditions are implemented as ‘averaged’ diseases, regardless of how much their vision or functioning is affected by the disease. In this work, we reported that longitudinal VA decline however has statistically significant and even clinically important impact on HRQoL. Comparing the loss of HRQoL associated with various health problems helps to put in perspective the potential impact achievable by the development of treatments for these conditions.

There are also potential limitations in our study. We were unable to assess the impact of other types of visual impairment, such as diminishing visual field, on HRQoL. The eye examination was carried out by a general practitioner, rather than an ophthalmologist, which can be considered as a weakness. However, it was necessary due to the large sample size and the complex study design. Another limitation was that we had to combine comorbidities into rather large groups (e.g., heart disease or psychiatric disorder) when estimating their impact on HRQoL, as new diagnoses during the 11-year follow-up are scarce for many specific diseases, such as psychosis. Moreover, we did not have the data available to differentiate disease types or severities, for example, specific cancer types, and had to account them as homogenous groups, regardless of the potential heterogeneity of specific diseases. Also, in the present analysis, we examined the general visual function and did not evaluate impact of specific vision-impairing diseases separately. For instance, age-related macular degeneration and glaucoma have been previously associated with declining QoL [57, 58]. In the future analyses it would be beneficial to evaluate and compare HRQoL impact of disease-specific HRQoL impacts in longitudinal setting. The long duration of the follow-up might also cause problems, as there has been progression on the therapies for various conditions. Also, the diagnostics have developed, meaning that a growing proportion of diseases are being diagnosed even symptomless.

Concerning EQ-5D and 15D index scores, we included only study participants with complete information collected in each assessment. The proportion of study participants with complete HRQoL data and insufficient VA-data was quite low, ranging from 0.4 to 0.8%, and therefore potential bias resulted by it is also likely to be low. Taking the loss to follow-up into account, it needs to be acknowledged that the proportion of study population with complete information from both time points is somewhat lower. Even so, the study sample remains large (4703 participants) and representative. Additionally, the study population was predominantly Finnish, and the results may not be applicable to other countries and ethnicities. However, the comparability is somewhat improved by our use of UK time-trade-off weights for EQ-5D instead of the Finnish preference weights.

Although the findings of the present study are based on large, representative Finnish population-based samples, more detailed analyses with large population-based study samples are required to validate the generalizability of these results into other settings as well. Moreover, additional studies with over 10 years of follow-up are necessary to ascertain the impact of declining vision on HRQoL.

## Conclusions

Our findings suggest that declining vision significantly affects quality of life even before the diagnosis of severe vision loss, visual impairment, or blindness. This encourages developing new or enhancing the existing treatment options aiming to improve vision, stop progression of eye diseases or at least postpone the onset of visual impairment or declining visual acuity, and the associated loss of quality of life. Regardless of all the treatment options available, some patients with declining VA experience problems in their daily life. The importance of rehabilitation and social services in maintaining good HRQoL is indisputable to reduce the functional disability caused by VA loss. This study provides HRQoL data comparable to other health conditions and changes in them, which helps evaluating the best possible allocation of health-care resources.

## Electronic supplementary material

Below is the link to the electronic supplementary material.

Supplementary material 1 (DOCX 109 kb)

## Abbreviations

15D: 15-Dimensional questionnaire assessing Health-Related Quality of Life
BDI: Beck Depression Inventory
EQ-5D: 5-Dimensional questionnaire assessing Health-Related Quality of Life
HRQoL: Health-Related Quality of Life
LALES: The Los Angeles Latino Eye Study
MCIC: Minimum Clinically Important Change
NEI VFQ-25: The National Eye Institute Visual Function Questionnaire
QoL: Quality of Life
VA: Visual acuity
VRQoL: Vision-Related Quality of Life
