# Supplementary material for: Low vision status and declining vision decrease Health-Related Quality of Life: Results from a nationwide 11-year follow-up study
Source: Qual Life Res. 2019 Aug 10;28(12):3225–36. doi: 10.1007/s11136-019-02260-3 (PMC6863947; doi:10.1007/s11136-019-02260-3)
Supplement: Supplementary file 1 — Supplementary material 1 (DOCX 109 kb) [file 11136_2019_2260_MOESM1_ESM.docx]

**Supplementary material**


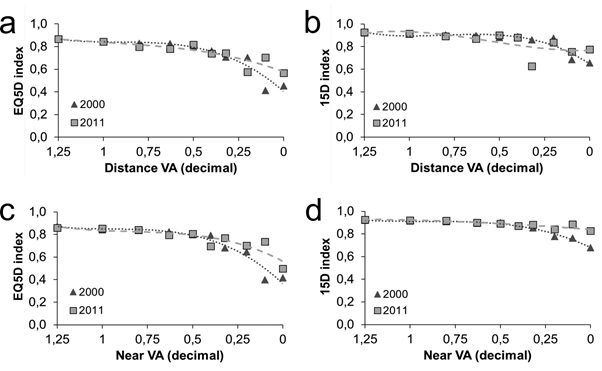
**Fig. S1**

Age and sex adjusted HRQoL index scores in relation to habitual distance (a, b) and near (c, d) VA in both time points. The y-axis represents the mean index value, where 1 is the best possible HRQoL. The x-axis represents VA in decimal equivalents from 1.25 (excellent vision) to 0 (blind). Dotted line represents the trend line for year 2000 and dashed line for year 2011.


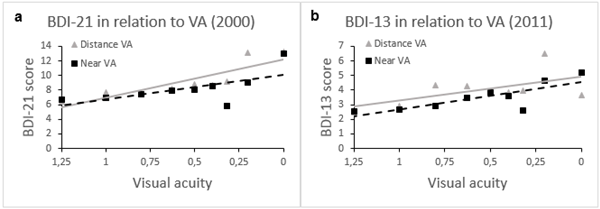


**Fig. S2**

Age-adjusted depression measured by BDI-questionnaire in relation to visual acuity. A: Scores from BDI-21 questionnaire in 2000. B: Scores from BDI-13 questionnaire in 2011

*Table S1. Stepwise-insertion regression analysis examining the changes in EQ-5D and 15D index value between 2000 and 2011, including only statistically significant factors (p<0.05).*

|  | **Change in EQ-5D (n=3068)** | | | **Change in 15D (n=3454)** | | |  |
| --- | --- | --- | --- | --- | --- | --- | --- |
|  | **B Coefficients** | **Beta Coefficients** | | **B Coefficients** | **Beta Coefficients** | |  |
| Constant | 0.403 ** | |  | 0.271 ** | |  | |
| Incident heart disease | -0.034 * | | -0.058 * | **-0.016 **** | | -0.070 ** | |
| Incident pulmonary disease | -0.028 * | | -0.039 * | **-0.025 **** | | -0.087 ** | |
| Incident vascular disease |  | |  | -0.013 * | | -0.044 * | |
| Incident musculoskeletal condition | -0.038 ** | | -0.105 ** | -0.007 * | | -0.049 * | |
| Incident hypertension | -0.031 ** | | -0.068 ** |  | |  | |
| Incident diabetes |  | |  | **-0.018 **** | | -0.067 ** | |
| Incident psychiatric disorder | -0.062 ** | | -0.071 ** | **-0.024 **** | | -0.069 ** | |
| Incident Parkinson disease |  | |  | **-0.076 **** | | -0.066 ** | |
| Incident cancer |  | |  | -0.012 * | | -0.040 * | |
| Change in Visual acuity (VA), compared to stable VA | | | | | | | |
| Distance VA declined | -0.063 ** | | -0.092 ** | **-0.032 **** | | -0.117 ** | |
| Near VA declined | -0.027 * | | -0.047 * | -0.012 * | | -0.054 * | |
| QoL index value in baseline | **-0.465 **** | | -0.421 ** | **-0.293 **** | | -0.312 ** | |
| R^2^ | 0.193 ** | | 0.191 ** | 0.131 ** | | 0.128 ** | |

*VA was considered improved or declined if difference of at least 2 lines in the Snellen eye-chart was observed between the time points. The unstandardized B coefficients show the magnitude of the impact on HRQoL while the standardized Beta coefficients allow the comparison of the explanatory variables with each other. Clinically meaningfully B coefficients are* *bolded (≥ 0.07 for EQ-5D and ≥ 0.015 for 15D [34, 35]). It should be noted that B regression coefficients represented in the table are independent and additive, meaning that if an individual experience a e.g. decline in both near and distance VA, the HRQoL impacts of both need to be considered (added together).*

** denotes statistical significance with p < 0.05*

*** denotes statistical significance with p < 0.001*

*Table S2. Stepwise-insertion regression analysis examining the change in 15D vision dimension between 2000 and 2011, including only statistically significant factors (p<0.05)*

|  | **Beta Coefficients** | **Sig.** | |  |
| --- | --- | --- | --- | --- |
| Constant | 0.679 | | <0.001 | |
| Incident pulmonary disease | -0.042 | | 0.003 | |
| Incident diabetes | -0.034 | | 0.017 | |
| Change in Visual acuity (VA), compared to stable VA | | | | |
| Distance VA declined | -0.193 | | <0.001 | |
| Near VA declined | -0.152 | | <0.001 | |
| 15D Vision dimension value in baseline | -0.492 | | <0.001 | |
| Adjusted R^2^ | 0.292 | | <0.001 | |
| *VA was considered improved or declined if difference of at least 2 lines in the Snellen eye-chart was observed between the time points.* *Only the standardized Beta coefficients reported to allow the comparison between the explanatory variables.* | | | | |
